# Real-world evidence on treatment pattern, effectiveness, and safety of blinatumomab in Chinese patients with B-cell acute lymphoblastic leukemia

**DOI:** 10.1007/s10637-024-01435-1

**Published:** 2024-04-25

**Authors:** Huifen Zhou, Xiaoxia Wu, Zhen Yang, Shenqi Lu, Xinhui Zhang, Xiaofei Yang, Suning Chen, Depei Wu, Miao Miao

**Affiliations:** Department of Hematology, The First Affiliated Hospital of Soochow University, Jiangsu Institute of Hematology, National Clinical Research Center of Hematologic Diseases, No 188, Shizi Street, Suzhou, Jiangsu 215006 China

**Keywords:** B-ALL, Blinatumomab, Newly diagnosed B-ALL, Relapsed/refractory B-ALL

## Abstract

Blinatumomab is efficacious in patients with B-cell acute lymphoblastic leukemia (B-ALL), yet limited real-world data exists in this context. This retrospective study provided real-world data on the treatment pattern, effectiveness, and safety of blinatumomab in Chinese patients with newly diagnosed (ND) and relapsed/refractory (R/R) B-ALL. Patients with B-ALL who received at least one dose of blinatumomab in frontline or R/R settings between August 2021 and June 2023 were included. The primary outcome was the treatment pattern of blinatumomab. Key secondary outcomes included complete remission (CR)/CR with incomplete blood cell recovery (CRi) rate, minimal residual disease (MRD) negativity, median event-free survival (EFS), and safety. The study included 96 patients with B-ALL; 53 (55.2%) patients were in the ND group and 43 (44.8%) patients were in the R/R group. The median treatment duration was one cycle (range: 1–5). Most patients underwent chemotherapies, allo-HSCT, or experimental CAR-T following blinatumomab. The ND patients using blinatumomab induction therapy achieved 100% CR/CRi rate; 87.2% achieved MRD negativity within two cycles of blinatumomab. In R/R re-induction patients, the CR/CRi rate was 50%; MRD negativity rate was 64.2%. In R/R patients using blinatumomab for consolidation, MRD negativity rate was 90.9%. The median EFS was not reached in both ND and R/R patients; 1-year EFS rate was 90.8% (95% CI: 67%, 97%) and 55.1% (95% CI: 30%, 74%), respectively. Grade ≥ 3 adverse events were observed in 12.5% patients. Blinatumomab was found to be effective with a tolerable safety profile in real world setting.

**Supplementary Information:**

The online version contains supplementary material available at 10.1007/s10637-024-01435-1.

## Introduction

B-cell acute lymphoblastic leukemia (B-ALL), constitutes 85% of ALL cases [1] and poses a complex treatment challenge, [2] traditionally relying on chemotherapy. Approximately 85% to 90% of newly diagnosed (ND) patients go into remission after the induction chemotherapy [3]. However, most patients with B-ALL relapse, the treatment of relapsed/refractory (R/R) B-ALL is challenging owing to chemo-resistance and chemotherapy-related side effects, causing a major burden on patients and their families [4].

Conventional salvage chemotherapy yields modest response rates (20–40%) and short-lived remission for R/R patients [5–7], with allogeneic hematopoietic stem cell transplantation (allo-HSCT) being the main curative option. However, most patients with R/R B-ALL cannot achieve complete remission (CR) with the salvage therapies and are not eligible for transplantation [8, 9].

In recent years, treatment for adult B-ALL, particularly in the R/R setting, has seen significant advancements driven by the development of immunotherapies. Blinatumomab, a bispecific T-cell engager (BiTE), has shown promise in enhancing B-ALL prognosis [10]. Based on the results from the TOWER [11] and BLAST [12] studies, currently, blinatumomab stands out as the first globally approved single-agent immunotherapy for treating R/R B-ALL and B-ALL in the first or later CR with a minimal residual disease (MRD) of ≥ 0.1% [13]. Further, the clinical studies of blinatumomab in the frontline treatment of B-ALL with an intent to enhance the depth of remissions, prevent relapses, and reduce toxicity have also shown encouraging results in ND B-ALL patients [14].

Although multiple studies have demonstrated the efficacy and safety of blinatumomab, there are limited data on the efficacy and safety of blinatumomab in the treatment of B-ALL, especially for ND patients, outside the clinical trials in China, i.e. in the real-world setting which encompasses the clinical practice of the treatment of B-ALL in China. Hence, we retrospectively collected data of patients with ND and R/R B-ALL treated with blinatumomab in our center to provide the real-world evidence that includes the clinical evidence about the usage and potential benefits and risks of blinatumomab treatment derived from the analysis of real-world data in our study.

## Methods

### Study design and patient population

In this retrospective observational study, data on B-ALL patients who received at least one dose of blinatumomab in frontline or R/R settings between August 2021 and June 2023 were collected from electronic medical data of the Department of Hematology of the First Affiliated Hospital of Soochow, China. Diagnostic criteria for B-ALL was based on the World Health Organization (WHO) classification criteria for malignant tumors of the hematopoietic system and lymphoid tissues [15]. Patients with R/R B-ALL were those who failed to achieve CR after the induction therapy and those with ≥ 5% blasts in the bone marrow or extramedullary relapse after documented CR by the National Comprehensive Cancer Network [16].

### Treatment and data collection

Patients ≥ 45 kg received a fixed blinatumomab dose (9–28 μg/day), while those < 45 kg received a dose based on body surface area (5–15 μg/m^2^/day). Infusion reaction and cytokine release syndrome (CRS) risks were mitigated with oral dexamethasone (20 mg for adults, 5 mg/m^2^ with a maximum 20 mg for pediatric patients) pre-treatment and before dose escalation. Demographic and clinical data were obtained from medical records, with treatment-related details documented at initial and follow-up visits. All cases were followed up by telephone, and information was acquired on the next hospital visit for the treatment. Patients were followed up till March 2023.

### Outcomes

The primary outcome of the study was the treatment pattern of blinatumomab therapy, targeting diverse populations and considering treatment duration. The secondary outcomes included CR/CR with incomplete blood cell recovery (CRi) rate, MRD negativity (i.e., complete MRD remission, defined as MRD < 0.01%) rate, event-free survival (EFS) and 1-year EFS rate, overall survival (OS) and 1-year OS rate, relapse-free survival (RFS) and 1-year RFS rate, duration of response (DOR) and adverse events (AEs). Bone marrow examination determined CR/CRi, MRD remission, disease progression, and disease recurrence. CR was defined as no evidence of extramedullary disease, ≤ 5% blasts in bone marrow, full recovery of absolute neutrophil count (ANC; ≥ 1.0 × 10^9^/L), and platelet count (≥ 100 × 10^9^/L) [17]. CRi was defined as CR but with incomplete recovery of peripheral blood counts (ANC < 1.0 × 10^9^/L) or platelet count (< 100 × 10^9^/L). MRD-negative or MRD remission was defined as < 1 × 10^−4^ leukemia cells detected by flow cytometry after treatment, without extramedullary infiltration of the central nervous system. EFS measured time from first blinatumomab infusion to first relapse, CR/CRi or death. Patients who failed to achieve CR/CRi within 12 weeks of treatment initiation were considered treatment failures and assigned an EFS duration of one day. OS was time from the initiation of blinatumomab to death from any cause. RFS was time from the date of CR/CRi to relapse or death, whichever occurred first. DOR measured time from the date of response (CR/CRi, MRD remission) to progression or death due to any reason, whichever occurred earlier. The safety of blinatumomab was evaluated based on AEs that were graded according to the Common Terminology Criteria for Adverse Events (CTCAE) 5.0 criteria [18].

### Statistical analyses

Demographic and patient characteristics were summarized by descriptive statistics. Continuous variables were summarized by n, mean, standard deviation, median, and ranges. Categorical variables were summarized by counts and percentages for each category. Proportion was reported for CR/CRi rate, and MRD negativity. Kaplan-Meier method was used to estimate time to event endpoints (i.e. EFS, OS, and DOR) curves, corresponding quantiles (including the median), and survival rates at selected timepoints. A two-sided 95% confidence intervals (CIs) of the median, if estimable, were constructed with generalized Brookmeyer and Crowley method, whereas the 95% CIs for survival rates at selected timepoints were calculated based on Greenwood’s formula.

## Results

### Baseline characteristics before blinatumomab initiation

A total of 96 B-ALL patients were included in the study; 53 (55.2%) were in the ND group and 43 (44.8%) were in the R/R group (Table 1). Among R/R patients, 18 (18.8%), 12 (12.5%), and 13 (13.5%) patients had 1, 2, and ≥ 3 lines of prior treatment, respectively.

**Table 1:** Baseline demographics and clinical characteristics of patients with B-ALL

**Characteristics**	**N = 96**
**Newly diagnosed, n (%)**	53 (55.2)
**Relapsed/refractory, n (%)**	43 (44.8)
**Age, years, median (range)**	34 (11, 67)
Newly diagnosed	36 (13, 67)
Relapsed/refractory	32 (11, 64)
< 35 years, n (%)	50 (52.1)
35 to < 55 years, n (%)	35 (36.5)
≥ 55 years, n (%)	11 (11.5)
**Sex, n (%)**	
Male	53 (55.2)
Female	43 (44.8)
**WBC (×10^9/L), mean (SD)**	61.4 (120.7)
**Hemoglobin count (×g/L), mean (SD)**	88.7 (34.5)
**Platelets count (×10^9/L), mean (SD)**	75.4 (69.9)
**Philadelphia chromosome, n (%)**	
Ph-	67 (69.8)
Ph+	29 (30.2)
**Bone marrow tumour burden at first time diagnosis, n (%)**	
< 50% blasts	10 (10.4)
≥ 50% blasts	78 (81.2)
**Central nervous system leukemia, n (%)**	
Yes	5 (5.2)
No	91 (94.8)
**Chemo-intolerable, n (%)**	
Yes	7 (7.3)
No	89 (92.7)
**Previous treatment lines, n (%)**	
0	53 (55.2)
1	18 (18.6)
2	12 (12.5)
≥ 3	13 (13.5)
**Prior HSCT, n (%)**	
Yes	12 (12.5)
No	84 (87.5)
**Prior CAR-T, n (%)**	
Yes	10 (10.4)
No	86 (89.6)
**Previous inotuzumab treatment, n (%)**	
No	96 (100)
**Refractory to the last treatment, n (%)**	
Yes	16 (16.7)
No	80 (83.3)
**Disease status on starting blinatumomab, n (%)**	
Response (CR)	52 (54.2)
Refractory (non-CR)	15 (15.6)
Status unknown	29 (30.2)
**MRD status on starting blinatumomab, n (%)**	
Positive (≥ 0.01%)	29 (30.2)
Negative (< 0.01%)	23 (24.0)
Status unknown	44 (45.8)
**Patients with abnormal genetic features, n (%)**	
t[4;11] or other KMT2Ar	5 (5.2)
t[12;21][p13.2;q22.1]/TEL-AML1 [ETV6-RUNX1]	7 (7.3)
t[1;19][q23;p13.3]/E2A-PBX1[TCF3-PBX1]	7 (7.3)
Hyperdiploid	6 (6.3)
Hypodiploid	1 (1)
Complex karyotype	2 (2.1)
IKZF1 deletion	6 (6.3)
Ph-like	6 (6.3)
PAX5	9 (9.4)

*B-ALL* B-cell acute lymphoblastic leukemia, *CR* complete response, *CAR-T* chimeric antigen receptor T-cell therapy, *HSCT* hematopoietic stem cell transplantation, *Ph* Philadelphia chromosome, *SD* standard deviation, *WBC* white blood cell

The median age of the overall cohort was 34 (range: 11–67) years; 55.2% were males. The median age of the ND and R/R groups was 36 (range: 13–67) years and 32 (range: 11–64) years, respectively. Overall, 29 (30.2%) patients were Ph+ , 49 (51.0%) patients had abnormal genetic features and 5 (5.2%) patients had central nervous system invasion at baseline. Further, 10 (10.4%) patients had previously undergone chimeric antigen receptor T-cell therapy (CAR-T), whereas 12 (12.5%) patients had received allo-HSCT (Table 1).

### Concurrent therapies before blinatumomab treatment

Of the 96 enrolled patients, 53 received frontline treatment (blinatumomab for first-line remission induction: n = 6; first-line consolidation: n = 39; unknown: n = 8), and 43 received treatment in R/R setting (blinatumomab for re-induction: n = 28; R/R consolidation: n = 14; unknown: n = 1). Among 52 patients (including 38 ND and 14 R/R), who used blinatumomab for consolidation, 44 were MRD-positive before blinatumomab initiation (33 ND and 11 R/R).

In the ND group, 32 and 21 patients were Ph- and Ph+ , respectively. Among 32 ND Ph- patients, 22 received short-term (3–7 days) low intensity chemotherapy before blinatumomab initiation, of whom 10 (45.4%) patients achieved CR after low intensity chemotherapy (Supplementary Table 1). Among 10 patients receiving chemotherapies induction and consolidation before blinatumomab initiation, all (100%) were in the CR state at blinatumomab initiation. Among 21 ND Ph+ patients, 15 patients received tyrosine kinase inhibitors (TKIs) with or without low intensity chemotherapies before blinatumomab initiation and 12 of them achieved CR (80%) at the start of blinatumomab. Six ND Ph+ patients received chemotherapy induction and consolidation along with TKIs before blinatumomab initiation and all of them (100%) achieved CR at blinatumomab initiation (Supplementary Table 1).

In the R/R group, 35 patients were Ph- and 8 patients were Ph+ . Among 35 R/R Ph- patients, 14 patients received blinatumomab directly at relapse. Another 13 patients received low intensity chemotherapy before blinatumomab initiation, of whom 4 (30.8%) achieved CR. The remaining 8 R/R Ph- patients received high intensity chemotherapies and/or CAR-T and/or transplantation before blinatumomab initiation. All except 1 patient achieved CR (Supplementary Table 1). The 8 R/R Ph+ patients received TKIs at blinatumomab initiation; 5 began blinatumomab at relapse. Of the other 3 patients, 2 received low-intensity chemotherapy with TKIs, and 1 received hyper-CVAD + TKI; all were in CR at blinatumomab initiation (Supplementary Table 1).

### Treatment pattern after starting blinatumomab

Most patients (66.7%) received 1 cycle of blinatumomab followed by 2 (20.8%), 3 (7.3%), 4 (4.2%), and 5 (1.0%) cycles of the drug**.** The median treatment duration was 1 cycle (range: 1–5) with a median follow-up of 205 (range: 15–652) days.

**Fig. 1:**
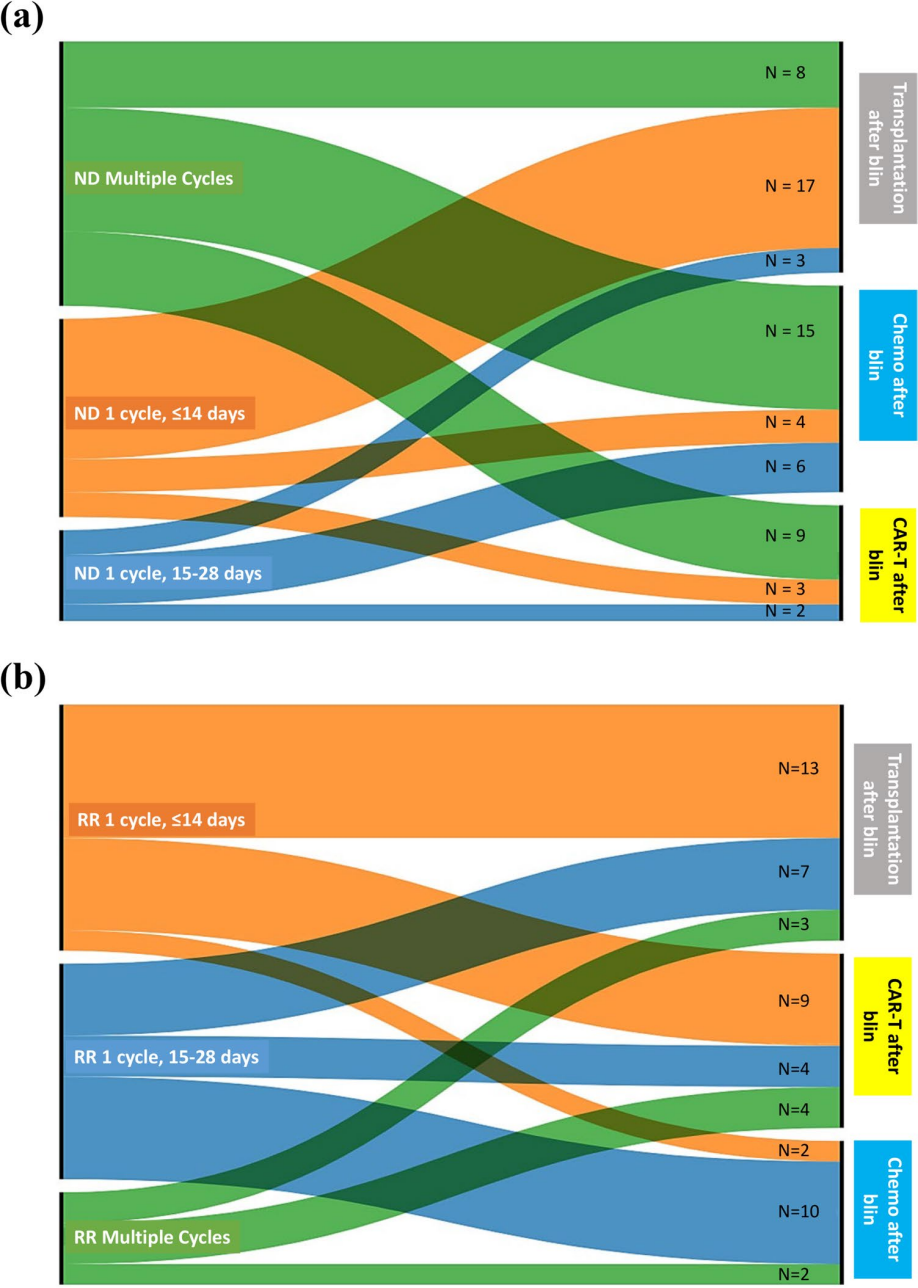
Treatment patterns in patients with – **a** newly diagnosed B-ALL and **b** Relapsed/refractory B-ALL. Abbreviations: B-ALL, B-cell acute lymphoblastic leukemia, Blin, blinatumomab; CAR-T, chimeric antigen receptor T-cell therapy; ND, newly diagnosed; R/R, relapsed/refractory

Figure 1 illustrates post-blinatumomab treatment options. ND patients with a shorter blinatumomab treatment duration (≤ 14 days) mostly opted allo-HSCT, whereas those with a longer blinatumomab treatment duration (15–28 days) favored chemotherapies post-blinatumomab (Fig. 1a). A similar trend emerged for patients undergoing 1 cycle versus multiple cycles, with single-cycle patients opting for transplantation and multiple-cycle patients choosing chemotherapies.

In the ND B-ALL group (53 patients), 14 received post-blinatumomab experimental CAR-T therapies. Among these, 7 exclusively received CAR-T until the last follow-up, 1 received CAR-T followed by chemotherapy, 3 received pre-CAR-T chemotherapies, 2 used CAR-T to bridge to allo-HSCT, and 1 received post-blinatumomab chemotherapy, followed by inotuzumab at first relapse, and subsequently experimental CAR-T and allo-HSCT. Among the 25 patients who received allo-HSCT after blinatumomab besides those mentioned previously, 14 received allo-HSCT directly, and 11 received additional chemotherapies before allo-HSCT (2 also received inotuzumab between chemotherapies and allo-HSCT, 1 received a reduced dose of inotuzumab, and the other 1 developed veno-occlusive disease requiring intensive care). Six patients received only chemotherapies after blinatumomab and 2 received chemotherapies along with inotuzumab. Only one patient discontinued blinatumomab because of AE and was treated with chemotherapy and inotuzumab.

In the R/R cohort, patients receiving shorter blinatumomab duration (≤ 14 days) and 1 cycle primarily underwent allo-HSCT and CAR-T. Those with 28-day blinatumomab mainly chose chemotherapies (Fig. 1b).

Among 30 R/R patients completing blinatumomab, 10 underwent direct allo-HSCT, 4 received direct experimental CAR-T, 3 used experimental CAR-T to bridge to allo-HSCT, 5 received chemotherapies alone (2 relapsed and received allo-HSCT and experimental CAR-T, respectively), 2 used chemotherapies to bridge to allo-HSCT; the remaining 6 patients did not receive other therapies while 3 of them received chemotherapies or allo-HSCT at the next relapse.

## Response and survival outcomes

### CR rate and MRD remission

In the frontline treatment, all those using blinatumomab for remission induction and first line unknown status achieved CR with a CR/CRi rate of 100% (14/14) after blinatumomab. In patients with available MRD records (n = 47), 87.2% (n = 41) achieved MRD negativity within 2 cycles of blinatumomab (Table 2). In the following cycles, 3 more patients achieved MRD remission with the overall MRD remission rate of 93.6% (44/47). Stratified by CR status at baseline, in 33 CR patients with available MRD records, 81.8% (27/33) achieved MRD remission within 2 cycles of blinatumomab and 90.9% (30/33) after the following cycles. Among 6 remission induction patients and 9 patients with unknown CR status, all (100%) achieved CR with MRD remission within the 2 cycles of blinatumomab.

**Table 2:** Efficacy outcomes of the patients

	**Blinatumomab treatment in the frontline setting (n = 53)**				**Blinatumomab treatment in the relapsed/refractory setting (n = 43)**			
	**Induction**	**Consolidation**		**Unknown status**	**Re-induction**	**Consolidation**		**Unknown status**
**Number of patients**	6	39		8	28	14		1
**CR/CRi rate**	100% (6/6)	NA		100% (8/8)	50% (14/28)	NA		100% (1/1)
**Complete MRD remission**	100% (6/6) overall	90.9% (30/33) overall		100% (8/8) overall	64.2% (9/14) overall	90.9% (10/11) overall		100% (1/1) overall
	87.2% (41/47) within 2 cycles of blinatumomab 93.6% (44/47) overall				73% (19/26) within 2 cycles of blinatumomab 76.9% (20/26) overall			

**EFS**								
Median (months)	NR (95% CI: NE, NE)				NR (95%CI: NE, NE)			
1 -year rate	90.8% (95% CI: 67.0%, 97.0%)				55.1% (95% CI: 30.0%, 74.0%)			
1 -year rate by CR status	100.0% (95% CI: NE, NE)		93.3% (95% CI: 61.3%, 99.0%)	NA	NE (95% CI: NE, NE)		66.8% (95% CI: 27.0%, 88.3%	NA
1 -year rate by Ph+	100.0% (95% CI: 100.0%, 100.0%)				NR (95% CI: NE, NE)			
1 -year rate by Ph-	83.0% (95% CI: 45.7%, 95.6%)				49.9% (95% CI: 20.2%, 73.9%)			

**RFS**								
Median (months)	NR (95% CI: NE, NE)		NR (95% CI: NE, NE)	NA	2nd line: NR (95% CI: NE, NE) 3rd line: 2.7 (95% CI: 1.0, NE)		NR (95% CI: NE, NE)	NA
1 -year rate	100% (95% CI: 100.0%, 100.0%)		93.8% (95% CI: 63.2%, 99.1%)	NA	NR (95% CI: NE, NE)		NR (95% CI: NE, NE)	NA
**Median OS**	NR (95% CI: NE, NE)				NR (95% CI: NE, NE)			
**1-year OS rate**	NR (95% CI: NE, NE)				70.9% (95% CI: 50.0%, 84.0%)			
**DOR**	NR (95% CI: NE, NE)				NR (95% CI: NE, NE)			

*CI* confidence interval, *CR/CRi* complete remission/ complete remission with incomplete blood cell recovery, *DOR* duration of response, *EFS* event-free survival, *MRD* minimal residual disease, *NA* applicable, *NE* not evaluable, *NR* not reached, *OS* overall survival, *Ph* Philadelphia chromosome, *RFS* relapse-free survival

In 28 R/R re-induction patients, CR/CRi rate was 50% (14/28) after blinatumomab treatment, with 64.2% (9/14) MRD negativity rate in CR patients. In 14 R/R patients with CR status before blinatumomab initiation, 90.9% (10/11) achieved MRD negative CR after blinatumomab. Overall, the MRD remission rate in R/R patients who achieved CR was 73% (19/26) within 2 cycles of blinatumomab treatment. One more patient achieved MRD remission in the following cycle (76.9%; 20/26; Table 2).

### EFS, RFS, DOR and OS

**Fig. 2:**
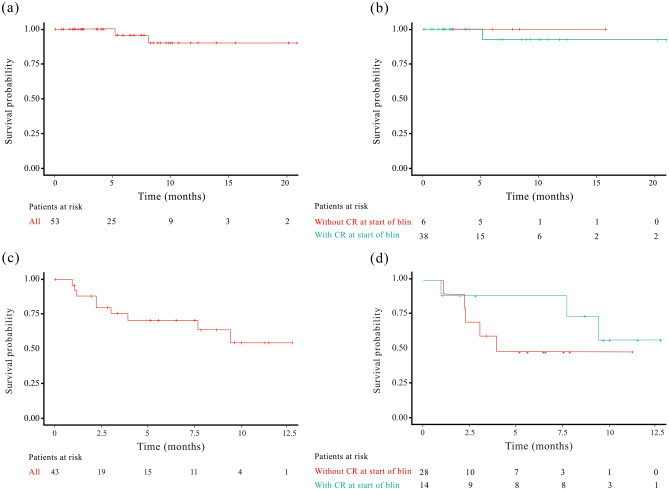
Kaplan-Meier curve for EFS in ND patients. **a** Overall and **b** Stratified by CR status before blinatumomab initiation and in R/R patients **c** Overall and **d** Stratified by CR status before blinatumomab initiation. Abbreviations: B-ALL, B-cell acute lymphoblastic leukemia; Blin, blinatumomab; CR, complete remission; EFS, event free survival; ND, newly diagnosed; R/R, relapsed/refractory

The median EFS was not reached (NR) in both frontline and R/R patients, and the 1-year EFS rate was 90.8% (95% CI: 67.0%, 97.0%) and 55.1% (95% CI: 30.0%- 74.0%), respectively (Fig. 2). The stratification of EFS by CR status at blinatumomab initiation showed a 1-year EFS rate of 93.3% (95% CI: 61.3%, 99.0%) and 100% (95% CI: not estimable [NE], NE) in the first-line consolidation and remission induction patients, respectively, whereas, R/R consolidation and re-induction patients showed a 1-year EFS rate of 66.8% (95% CI: 27.0%, 88.3%) and NE (95% CI: NE, NE), respectively (Table 2).

When stratified by Ph-chromosome status, the median EFS, without censoring for transplant and CAR-T, was NR in both Ph+ (95% CI: NE, NE) and Ph- (95% CI: 9.4, NE) B-ALL patients (Supplementary Fig. 1), and the 1-year EFS rate was 92.9% (95% CI: 59.1%, 99.0%) and 65.7% (95% CI: 41.8%, 81.7%) in Ph+ and Ph- B-ALL patients, respectively. The median EFS, with censoring for transplant and CAR-T, was also NR in both Ph+ (95% CI: 3.9, NE) and Ph- (95% CI: 7.7, NE) B-ALL patients, and the 1-year EFS rate was NR in Ph+ B-ALL patients and 48.0% (95% CI: 18.5%, 72.6%) in Ph- B-ALL patients.

On stratification by blinatumomab treatment line setting and Ph-chromosome status of B-ALL patients, the 1-year EFS (without censoring for transplant and CAR-T) was 100.0% (95% CI: 100.0%, 100.0%) in Ph+ ND patients and NR (95% CI: NE, NE) in Ph+ R/R patients receiving blinatumomab. The corresponding 1-year EFS rate was 83.0% (95% CI: 45.7%, 95.6%) in Ph- ND patients and 49.9% (95% CI: 20.2%, 73.9%) in Ph- R/R patients (Table 2).

The median RFS (without censoring for transplant and CAR-T) was NR in frontline blinatumomab remission induction patients and second-line re-induction R/R patients and 2.7 (95% CI: 1.0, NE) months in third-line R/R re-induction patients. Median EFS was NR in both frontline and R/R patients using blinatumomab for consolidation. The 1-year RFS rate was 100.0% (95% CI: 100.0% 100.0%) and 93.8% (95% CI: 63.2%, 99.1%) in frontline induction and consolidation patients, respectively, it was NR in both second-line and third-line R/R re-induction and consolidation patients (Table 2).

OS results in ND patients were NE. In R/R patients, median OS was NR and the 1-year OS rate was 70.9% (95% CI: 50.0%, 84.0%). Stratification of OS results by CR status in R/R patients showed a 1-year OS rate of 87.5% in consolidation patients and 69.9% in re-induction patients. The median DOR was NR in both frontline and R/R patients (Table 2).

### Safety

During blinatumomab treatment, AEs of any grade were reported in 19.8% patients (19/96) (Table 3). The most common AE of any grade was CRS (7/96; 7.3%) followed by infection (6/96; 6.2%). Grade ≥ 3 AEs were observed in 12.5% (12/96) of patients with infection (4/96; 4.2%) being the most common grade ≥ 3 AE followed by CRS (3/96; 3.1%). No deaths due to blinatumomab related AEs were reported. Two patients discontinued the blinatumomab treatment due to AEs, one patient had Grade 3 neurotoxicity, and the other one had grade 3 infusion reaction; both recovered after treatment discontinuation.

**Table 3:** Summary of AEs in all patients with B-cell acute lymphoblastic leukemia

	**Total number of patients, N = 96**		
	**Any grade**	**Grade 1–2**	**Grade ≥ 3**
**AEs, n (%)**	19 (19.8)	7 (7.3)	12 (12.5)
CRS	7 (7.3)	4 (4.2)	3 (3.1)
ICANS	1 (1)	0	1 (1)
Infection	6 (6.2)	2 (2.1)	4 (4.2)
Liver enzymes increase	1 (1)	0	1 (1)
Neutropenia	1 (1)	0	1 (1)
Others	3 (3.1)	1 (1)	2 (2.1)

*AEs* adverse events, *CRS* cytokine release syndrome, *ICANS* immune effector cell-associated neurotoxicity syndrome

During the follow-up period, in the ND group, 1 death was reported after 4 months post allo-transplantation due to transplantation related toxicity. In the R/R group, 13 patients discontinued blinatumomab treatment prematurely – 1 due to AE and 12 patients due to progressive diseases (PD), while 10 deaths were reported – 7 due to PD, and 1 each due to CRS induced by CAR-T, chemotherapy related toxicity, and chemotherapy related infection.

## Discussion

The treatment landscape of B-ALL has changed over recent years following the development of targeted therapies such as blinatumomab that is being studied extensively in clinical trials [11]. However, there is a paucity of comprehensive real-world studies of blinatumomab in B-ALL. Moreover, most of the available real-world studies have been conducted in Western countries while studies focusing China are very limited, where blinatumomab is mostly paid out-of-pocket. The current study retrospectively evaluated the real-world effectiveness and safety of blinatumomab in Chinese patients with ND and R/R B-ALL, as well as the treatment pattern of such an expensive agent that included different durations of treatment with blinatumomab, and the relationship between the duration of blinatumomab treatment and different treatment modalities used post-blinatumomab, i.e. chemotherapy, CAR-T, and transplantation in these patients. The results of the study showed the effectiveness of blinatumomab, leading to significant responses in both ND and R/R settings and tolerable safety profile in Chinese patients [19, 20]. In our study, patients with R/R B-ALL, who received blinatumomab for re-induction, showed a CR rate of 50% (14/28) which was higher than that reported in the TOWER multinational study (44%), which could be due to older age (41 years in the TOWER study vs 32 years in our study) and more heavily pre-treated patients with ~58% of patients receiving blinatumomab as ≥ second-line salvage therapy [11]. However, the MRD negativity observed in the current study was similar to that of the TOWER study (76%). Among 43 R/R patients receiving blinatumomab, 64% successfully bridged to allo-HSCT, showing a potential OS improvement. These observations were consistent with previous studies that showed allo-HSCT to be safe and effective after blinatumomab in patients with R/R B-ALL [21]. Notably, blinatumomab has been used for variable durations in our study because although the drug has been approved for the treatment of adult patients with R/R B-ALL in China, it is still not covered under the National Reimbursement Drug List (NRDL), and is not eligible for reimbursement through basic health insurance and patients have to bear the cost themselves. From our data, R/R patients using blinatumomab for a shorter duration (≤ 14 days) were mostly likely to bridge to allo-HSCT (54.0%), which is also a costly medical procedure adding to the overall treatment cost, or experimental CAR-T therapies (37.5%), which are commonly followed by allo-HSCT, thereby possibly trying to take the most advantage of blinatumomab in MRD eradication without adding too much to the overall treatment cost. ND patients, with longer blinatumomab cycles (multiple cycles, n = 24 [45.3%]), experienced lower financial burdens, choosing allo-HSCT less frequently (33.3%).

Frontline B-ALL treatment is crucial, aiming for disease remission using standard multidrug chemotherapy with a CR rate of 80–90% and 30–50% long-term survival [14, 22]. Blinatumomab, approved for R/R B-ALL, holds promise as an addition to frontline chemotherapy, potentially elevating response rates. In this study, median EFS was NR in frontline and R/R patients, but 1-year EFS was significantly higher in pretreated ND patients than R/R patients (90.8% vs 55.1%). ND patients treated with blinatumomab had higher CR rates than R/R patients (100% vs 50%), aligning with a previous Chinese study (100%) [23]. Further, Ph+ chromosome is the most common cytogenetic abnormality in B-ALL and is associated with poor outcomes [24]. Literature indicates that TKIs combined with chemotherapy may reduce its intensity without compromising efficacy, thus minimizing associated AEs [24, 25]. Studies are now ongoing with the combination of chemotherapy and TKIs or TKI alone along with blinatumomab in B-ALL. In a recent D-ALBA trial, ND Ph+ B-ALL patients were given the induction therapy with dasatinib followed by 2 cycles of blinatumomab resulting in improved molecular response rate from 29% at pre-blinatumomab level to 60% after the first cycle, which increased further after additional cycles of blinatumomab [26]. These results add to the growing evidence supporting the use of blinatumomab in the frontline setting in B-ALL. Further, in our study, the 1-year EFS rate was higher for ND Ph+ B-ALL patients treated with TKI-containing regimen followed by blinatumomab treatment regimen compared with ND Ph- B-ALL patients (100% vs 83.0%). A similar trend was observed for R/R patients with 1-year EFS NR in R/R Ph+ patients versus 49.9% in R/R Ph- patients, demonstrating that Ph+ may no longer be a poor prognostic factor in the era of TKIs and immunotherapies.

Allo-HSCT is recommended for high-risk patients within the first CR and for patients after the first relapse [27]. In our study, 53.1% (51/96) of blinatumomab-treated patients underwent transplantation. Only 15 (29%) transplanted patients were Ph+ , suggesting that blinatumomab and TKI treatment led to a lasting remission, potentially causing the transplant-sparing effect. This is crucial clinically, as transplantation, a complex procedure, is linked to potential complications [28]. Blinatumomab might serve as a potential transplantation alternative in Ph+ B-ALL, but further studies are needed to validate our findings.

The observed AEs align with blinatumomab's known safety profile [29]. CRS occurred in 7.6% (grades 1–2) and 3.1% (grade 3) of patients [11, 30]. In our study, 52 of 96 patients, 37 ND, and 15 R/R, received low-dose chemotherapy before blinatumomab, achieving a sustained good CR rate. Integrating targeted agents into frontline regimens, rather than reserving them for R/R scenarios, can enhance B-ALL outcomes. Considering blinatumomab's success in R/R B-ALL, studies explored its addition to frontline chemotherapy. Richard-Carpentier et al. combined blinatumomab with cyclophosphamide, vincristine, doxorubicin, and dexamethasone for adults with ND Ph- B-ALL, achieving 100% CR and 96% MRD negativity (sensitivity 0.01%). Eight patients (30%) underwent allo-HSCT, with 1-year relapse-free survival and OS at 76% and 89%, respectively [31]. Similarly, incorporating blinatumomab with dasatinib for frontline Ph+ B-ALL increased the overall response rate to 80% after 4 cycles, with 1-year OS and disease-free survival at 94% and 88%, respectively [32].

Our study may be limited by the nature of the design as an observational study with unimpeded access to medical data and possibilities of selection bias. Further, being conducted at a single center with a relatively small population. Substantiation of these results in a larger patient population, especially for patients with ND Ph+ B-ALL will provide additional insight into the applicability of these findings. Despite these limitations, the study unequivocally demonstrated the effectiveness and safety of blinatumomab in patients with ND and R/R B-ALL confirming a new option in the treatment armamentarium for Chinese B-ALL patients.

## Conclusion

In conclusion, this retrospective study affirms blinatumomab's effectiveness and tolerable safety in treating Chinese B-ALL patients, both in frontline and R/R scenarios. Our findings support the growing evidence favoring blinatumomab's integration with chemotherapy in frontline B-ALL therapy. Moreover, the study highlights comparable efficacy and safety of blinatumomab in Chinese B-ALL patients, pre-treated with low-dose chemotherapy, to global clinical trial outcomes. Given the limited real-world data, further studies are essential to guide physicians in making informed clinical decisions and enhancing patient outcomes in everyday clinical practice in the treatment of B-ALL in China.

### Supplementary Information

Below is the link to the electronic supplementary material.

Supplementary file1 (DOCX 110 kb)

## Data Availability

No datasets were generated or analysed during the current study.
