# Supplementary material for: Real-world evidence on treatment pattern, effectiveness, and safety of blinatumomab in Chinese patients with B-cell acute lymphoblastic leukemia
Source: Invest New Drugs. 2024 Apr 25;42(3):299–308. doi: 10.1007/s10637-024-01435-1 (PMC11164718; doi:10.1007/s10637-024-01435-1)
Supplement: Supplementary file 1 — Supplementary file1 (DOCX 110 kb) [file 10637_2024_1435_MOESM1_ESM.docx]

Real-world evidence on treatment pattern, effectiveness, and safety of blinatumomab in Chinese patients with B-cell acute lymphoblastic leukemia

Huifen Zhou^1^, Xiaoxia Wu^1^, Zhen Yang^1^, Shenqi Lu^1^, Xinhui Zhang^1^, Xiaofei Yang^1^, Suning Chen^1^, Depei Wu^1^, Miao Miao^1*^

^1^Department of Hematology, The First Affiliated Hospital of Soochow University, Jiangsu Institute of Hematology, National Clinical Research Center of Hematologic Diseases, 215000, Suzhou, China

*Correspondence:

Miao Miao

Department of Hematology

The First Affiliated Hospital of Soochow University, Jiangsu Institute of Hematology, National Clinical Research Center of Hematologic Disease. No 188, Shizi Streat, Suzhou, Jiangsu, 215006, China

Email ID: [mm85124@sina.com](mailto:mm85124@sina.com)

Contact no: +86-13962194350

Supplementary Table 1. Concurrent therapies before blinatumomab treatment

|  | **Number of patients** | **Response at the start of blinatumomab** | **Response after blinatumomab** |
| --- | --- | --- | --- |
| **Newly diagnosed Ph-** | **32** |  |  |
| Low intensity chemo regimen used before blinatumomab^†^ | 22 | CR: n=10 (45.4%); MRD negative: n=2  CR not achieved: n=6 (27.2%)  CR status unknown: n=6 (27.2%) | CR: n=21 (95.5%); MRD negative: n=21  CR status unknown: n=1 (4.5%) |
| Chemo induction and consolidation | 10 | CR: n=10 (100%); MRD negative: 7 | CR: n=7 (70%); MRD negative: n=7  CR status unknown: n=3 (30%) |
| **Newly diagnosed Ph+** | **21** |  |  |
| TKI +/- low intensity chemo used before blinatumomab^‡^ | 15 | CR: n=12 (80%); MRD negative: n=7  CR status unknown: n=3 (20%) | CR: n=14 (93.3%); MRD negative: n=12  CR status unknown: n=1 (6.7%) |
| Chemo induction and consolidation | 6 | CR: n=6 (100%); MRD negative: 5 | CR: n=5 (83.3%); MRD negative: 4  CR status unknown: n=1 (16.7%) |
| **Relapsed/refractory Ph-** | **35** |  |  |
| Low intensity chemo regimen used before blinatumomab^§^ | 13 | CR: n=4 (30.8%); MRD negative: n=1  CR not achieved: n=8 (61.5%)  CR status unknown: n=1 (7.7%) | CR: n=10 (76.9%); MRD negative: n=7  CR not achieved: n=3 (23.1%) |
| High intensity chemotherapy and/or CAR-T and/or transplantation | 8 | CR: n=7 (87.5%); MRD negative: n=1  CR not achieved: n=1 (12.5%) | CR: n=7 (87.5%); MRD negative: n=6  CR not achieved: n=1 (12.5%) |
| Direct blinatumomab at relapse | 14 | CR not achieved: n=14 (100%) | CR: n=6 (42.9%); MRD negative: n=4  CR not achieved: n=8 (57.1%) |
| **Relapsed/refractory Ph+** | **8** |  |  |
| Low intensity chemo + TKI^¶^ | 2 | CR: n=2 (100%) | CR: n=2 (100%); MRD negative: n=1 |
| High intensity chemotherapy and TKI^\|\|^ | 1 | CR: n=1 (100%) | CR status unknown: n=1 (100%) |
| Direct blinatumomab at relapse | 5 | CR not achieved: n=5 (100%) | CR: n=2 (40%); MRD negative: n=1  CR not achieved: n=2  CR status unknown: n=1 |

^†^Regimes used were IVP, VP, VDCP, IV, and DEX alone. ^‡^The regimens included: DEX + TKI, IVP + TKI, VP + TKI, VAF, MOP + TKI, IOP + TKI, and TKI alone. TKIs used were dasatinib, flumatinib and olverembatinib. ^§^The regimens used were: CLAG, VP, IVP, IV, CAVen, hydroxyurea + cyclophosphamide + mitoxantrone, MVP, CIP, hydroxyurea + dexamethasone + cyclophosphamide, and DEX alone. ^¶^VA + olverembatinib; CP + olverembatinib. ^||^Hyper-CVAD + flumatinib

Abbreviations: CAR-T, chimeric antigen receptor T-cell therapy; CAVen, cyclophosphamide + adriamycin + venetoclax; CIP, idarubicin + cisplatin + prednisone; CLAG, cladribine + cytarabine + G-CSF + mitoxantrone; CVAD, cyclophosphamide, vincristine, doxorubicin, and dexamethasone; CR, complete remission; DEX, dexamethasone; G-CSF, granulocyte colony stimulating factor; IOP, idarubicin + vinorelbine + prednisone; IV, idarubicin + vindesine; IVP, idarubicin + vincristine + prednisone; MOP, mitoxantrone + vindesine + prednisone; MRD, minimal residual disease; MVP, methotrexate-VP; Ph, Philadelphia chromosome; TKI, tyrosine kinase inhibitor; VA; VAF, venetoclax + azacitidine; VAF, venetoclax + azacitidine + flumatinib; VDCP, vincristine + daunorubicin + cyclophosphamide + prednisone; VP vincristine + prednisone; TKI, tyrosine kinase inhibitor


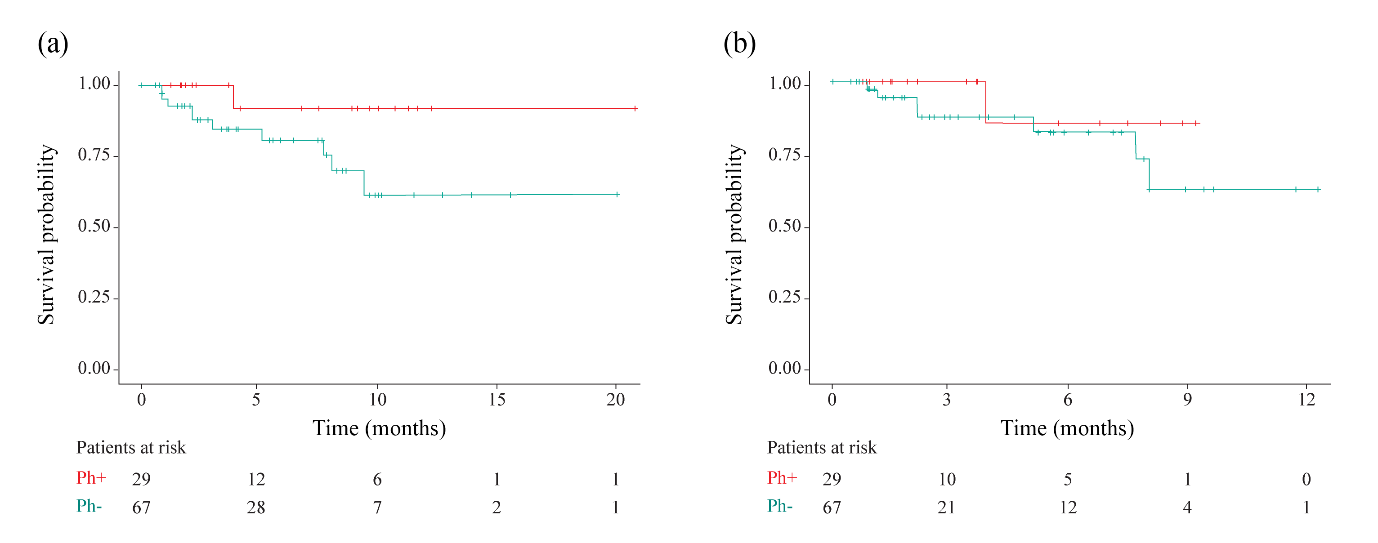


Supplementary **Fig. 1** Kaplan-Meier curve for EFS in patients with B-ALL patients stratified by Ph-chromosome status. (A) Not censored for transplantation or CAR-T and (B) Censored for both transplantation and CAR-T

Abbreviations: B-ALL, B-cell acute lymphoblastic leukemia; Blin, blinatumomab; CAR-T, chimeric antigen receptor T-cell therapy; EFS, event free survival; Ph, Philadelphia chromosome
